# A novel endovascular treatment for true ophthalmic aneurysms: A case report

**DOI:** 10.3389/fopht.2022.940479

**Published:** 2022-09-30

**Authors:** Taylor Furst, Thomas K. Mattingly, Zoë R. Williams, Derrek Schartz, Matthew T. Bender

**Affiliations:** ^1^ Department of Neurological Surgery, University of Rochester Medical Center, NY, Rochester, United States; ^2^ Department of Ophthalmology, University of Rochester Medical Center, NY, Rochester, United States; ^3^ Department of Imaging Sciences, University of Rochester Medical Center, NY, Rochester, United States

**Keywords:** ophthalmic artery, aneurysm, endovascular, flow diversion, coiling, balloon test occlusion, angiography

## Abstract

**Introduction:**

Cerebral aneurysms located along the internal carotid artery at the origin of the ophthalmic artery can be treated through open surgery or endovascular technique. The former affords more certainty of aneurysm obliteration, while the latter poses less risk to vision. Flow diversion is an increasingly accepted treatment for side-wall carotid aneurysms, although location at the branch point of the ophthalmic artery is known to moderate occlusion outcomes.

**Case presentation:**

We present a case of a middle-aged female patient with a morphologically irregular 4-mm ophthalmic artery aneurysm (OphA) and a smaller superior hypophyseal artery (SHA) aneurysm whose successful and uncomplicated obliteration by flow diversion with adjunctive coiling was predicted *via* a balloon test occlusion (BTO). BTO was employed prior to stent placement to confirm a) ophthalmic artery distal collateralization with external carotid artery (ECA) branches and b) preserved arterial flow in the retina visualized *via* fundoscopy. At 1 year following angiography, the patient had no postoperative deficits and benefitted from complete occlusion of the OphA and SHA.

**Conclusion:**

OphAs constitute a complex surgical disease that is historically associated with high visual morbidity. We present a novel advanced endovascular technique of BTO followed by flow diversion with adjunctive coiling that successfully obliterated an OphA while preserving vision.

## Introduction

Cerebral aneurysms cause three general categories of symptoms, each of which can have ophthalmologic manifestations: 1) mass effect, as in the case of a giant petrocavernous aneurysm invading the cavernous sinus and causing ophthalmoplegia, anterior communicating artery aneurysms compressing the optic chiasm causing visual deficits, and ophthalmic artery aneurysms compressing the optic nerve; 2) ischemia from large aneurysms with turbulent flow and vascular stasis that can cause embolic stroke in the occipital lobe, among other territories; and, lastly, 3) subarachnoid hemorrhage (SAH) with attendant vitreous hemorrhage causing vision loss, known as Terson Syndrome. These provide a rationale for elective treatment in some patients of unruptured cerebral aneurysms, which carry a rupture risk of approximately 1% per year (1–3).

Aneurysms arising from the proximal intracranial internal carotid artery are often generically called “paraophthalmic” but can more precisely be divided into clinoidal segment aneurysms proximal to the origin of the ophthalmic artery, medially projecting superior hypophyseal artery (SHA) aneurysms, and aneurysms from the base or dome of which the ophthalmic artery arises, which are called “true ophthalmic” aneurysms (OphA) (4). This last category warrants special consideration of treatment options because of potential visual morbidity.

Two broad categories of treatment options exist for cerebral aneurysms: open surgery and endovascular intervention, the latter accomplished through a variety of methods, including coils deployed inside the aneurysm and stents deployed across the aneurysm neck. What follows is a case presentation and novel balloon test occlusion (BTO) utilized to maximize aneurysm obliteration and vision preservation.

## Case presentation

**Figure 1:**
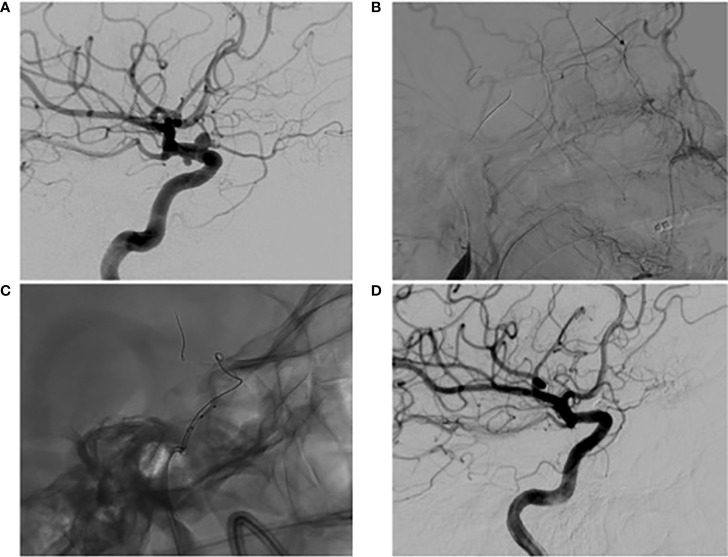
Digital subtraction angiography **(A)** pre-operative high magnification lateral view of left ICA angiography demonstrating a 4mm superiorly projecting OphA with associated SHA; **(B)** lateral view of left common carotid artery angiography during BTO revealing an occluded ICA with ECA to ophthalmic artery collateralization determined by the presence of choroidal blush (arrow); **(C)** high magnification lateral view of a jailed coiling microcatheter accessing the aneurysm; **(D)** lateral view of one year post-operative follow up left ICA angiography demonstrating obliteration of the OphA and SHA with preserved patency of the ophthalmic artery.

A middle-aged female current smoker presented neurologically intact with a severe headache. She was worked up for SAH with a negative lumbar puncture and MRI after the CT angiogram showed multiple morphologically irregular aneurysms located along the proximal ICA. Angiography then demonstrated a diseased vessel segment with a 4-mm morphologically irregular wide-necked OphA with an associated SHA aneurysm (**Figure 1**). Given no signs of sentinel hemorrhage, the patient was treated on an elective basis after extensive conversations regarding the operative plan, and rigorous informed consent was obtained. Treatment was recommended to the patient, as there is angiographic evidence of a morphologically irregular aneurysmal dome. Having an irregular morphology is associated with high rupture risk despite a low PHASES score, and this risk is heightened due to the patient’s smoking habits (5, 6). As such, a surgical plan was devised in an attempt to provide a cure while minimizing morbidity.

**Figure 2:**
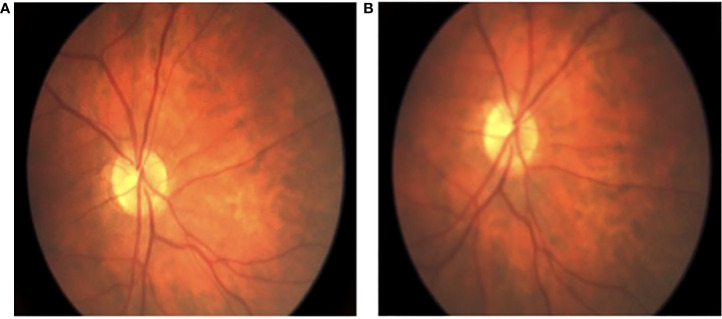
Intraoperative fundoscopic view of retinal perfusion. **(A)** fundoscopic view of baseline retinal arterial flow; **(B)** fundoscopic view of preserved retinal arterial flow after successful flow diverting stent deployment and adjunctive coiling.

The decision was made to perform endovascular flow diversion with possible adjunctive coiling pending the results of a novel balloon test occlusion protocol that was adapted from those previously described (7–9). The patient was started on dual antiplatelet therapy (DAPT) 2 weeks prior to treatment, and P2Y12 testing on the day of surgery confirmed clopidogrel responsiveness. Under general anesthesia and systemic heparinization with an activated clotting time (ACT) of >250, a 0.088″ Neuron Max guide catheter (Penumbra Inc., Alameda, CA, USA) was positioned into the common carotid artery. Ophthalmology then performed a baseline retinal examination (**Figure 2**). A single-lumen 7 mm × 7 mm TransForm super-compliant balloon (Stryker Neurovascular, Fremont, CA, USA) was introduced and inflated with a 50:50 mixture of contrast and saline at the level of the ophthalmic artery. Control angiography demonstrated no anterograde filling of the ophthalmic artery, while common carotid angiography showed choroidal blush *via* external carotid collaterals. Repeat retinal examination after 5 min of balloon inflation showed unchanged good retinal perfusion (**Figure 2**).

Having passed the balloon test occlusion, the risk of ophthalmologic complications was minimized, and other inherent risks of the procedure, including thrombo-embolic consequences, were felt to be comparable to those of flow diversion and coiling at other anatomic sites within the circle of Willis. Thus, we proceeded with flow diversion with adjunctive coiling using a single-intermediate technique as described elsewhere (10). A 0.0165″ inner diameter SL-10 45° microcatheter (Stryker Neurovascular, Fremont, CA, USA) and 0.027″ Phenom microcatheter (Medtronic, Minneapolis, MN, USA) were introduced through a 6F Sofia intermediate catheter (MicroVention Inc., Aliso Viejo, CA, USA). The coiling catheter was jailed inside the aneurysm dome, while a 4.5 mm × 15 mm Surpass Evolve (Stryker Neurovascular, Fremont, CA, USA) was deployed within the internal carotid artery across the aneurysm neck. Two coils were then deployed within the aneurysm. The dome was de-accessed, the coiling microcatheter was removed, and the TransForm balloon was reintroduced to angioplasty along the length of the stent.

The patient was visually and neurologically intact postoperatively and has remained that way. There were plans to continue DAPT for 6 months postoperatively. At that time, follow-up angiography was to be performed prior to transitioning to aspirin monotherapy. Due to illness and scheduling conflicts, however, follow-up angiography was instead performed 9 months post-procedure. At that time, angiography revealed a widely patent stent, anterograde flow through the ophthalmic artery, and obliteration of the OphA and SHA (**Figure 1**). The patient was then transitioned to aspirin monotherapy and has remained satisfied with her treatment. A further postoperative ophthalmological evaluation was not pursued, as the patient’s vision has remained at baseline.

An ophthalmological retinal evaluation was a novel addition to a BTO that was able to predict a positive visual outcome. Though the risks of BTO, including arterial injury, ischemic injury, and thrombo-embolic events, are not negligible, it provides critical information that allows the surgeon to provide aggressive treatment in an effort to achieve a cure. Additionally, since the described case is one of a diseased ICA segment (i.e., both an OphA and SHA), flow diversion was especially efficacious, as it provides a method to address the entire diseased segment in one treatment. The positive angiographic and clinical outcome presented above supports the use of this technique in cases of isolated OphAs as well.

**Figure 3:**
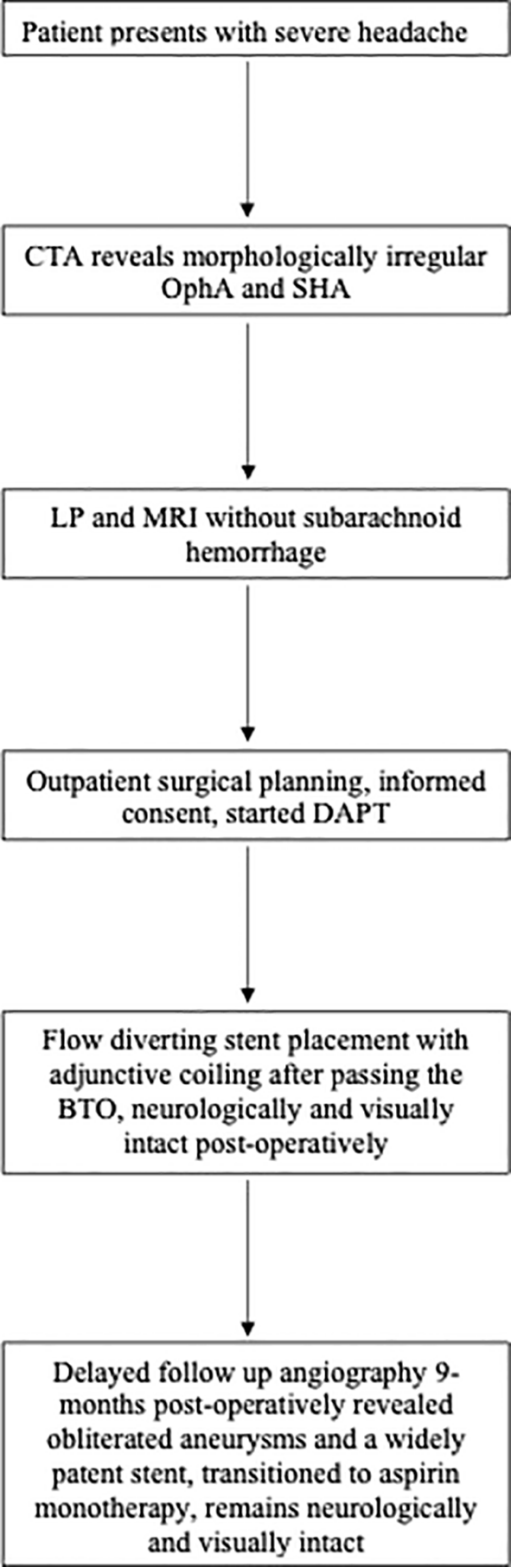
A representative timeline depicting the management of a morphologically irregular ophthalmic artery aneurysm. Computed tomography angiogram (CTA); Ophthalmic artery aneurysm (OphA); Superior hypophyseal aneurysm (SHA); Lumbar puncture (LP); Magnetic Resonance Imaging (MRI), Dual antiplatelet therapy (DAPT); Balloon test occlusion (BTO).

A timeline depicting the course of management and care can be found in **Figure 3**.

## Discussion

The tradeoff between the safety and efficacy of surgical and endovascular cerebral aneurysm treatment is accentuated in true ophthalmic aneurysms because of proximity to the optic nerve and location at a branch vessel. First detailed here is a balloon test occlusion of the ophthalmic artery before flow diversion with adjunctive coiling to identify ophthalmic artery collateralization *via* angiography and fundoscopy. This novel approach to BTO with an intraoperative fundoscopic evaluation of retinal perfusion can be utilized to identify OphAs that can be safely treated with curative endovascular techniques.

Flow diversion is an endovascular alternative to coiling in which a low-porosity braided stent situated across the aneurysm neck serves as a neoendothelialization scaffold to reconstruct the parent artery and gradually exclude the aneurysm from the circulation. Ophthalmic aneurysms are within the original indication of flow diverters (11). Their use has since expanded and become an integral part of an endovascular surgeon’s repertoire due to a similar complication profile, yet higher rates of complete occlusion when compared to coiling (12). A limitation to their use, however, includes aneurysms located at branch points, as they have diminished occlusion rates after single-modality flow diversion when compared to sidewall aneurysms (13). This is particularly true when the branch vessel supplies a poorly collateralized terminal circulation. This is the result of a persistent pressure gradient across the ostium of the aneurysm due to ongoing distal flow demands. Conversely, when distal collaterals are present, the competitive circulation neutralizes the pressure gradient across the stent, limiting aneurysm inflow and allowing thrombus formation within the aneurysm and endothelialization of the flow diverter.

It is important to identify aneurysms that are unlikely to occlude after single-modality flow diversion because the presence of a stent and the inherent need for DAPT complicate surgical salvage (14). Kan et al. (9) described seven cases of aneurysms that did not occlude with flow diversion and identified all as existing at branches supplying terminal circulations. The authors gave the traditional example of an aneurysm located at the origin of a fetal-type posterior cerebral artery, but also performed BTO of the ophthalmic artery. A BTO allowed the authors to identify an ophthalmic artery without collateral supply from the external carotid artery to predict the persistence of an associated OphA.

A variety of criteria have been used to decide whether a patient passes balloon test occlusion, including clinical examination in awake patients and electrographic *via* somatosensory or motor-evoked potentials (9, 15, 16). Collaborating with colleagues in the Ophthalmology Department, we evaluated the BTO based on angiographic evidence of external carotid artery (ECA) to ophthalmic artery collaterals and direct fundoscopic visualization of retinal perfusion without awakening of the patient. Others have described using visual evoked potentials and intraoperative awakening for visual testing during surgical clipping of an OphA, which could feasibly be used during endovascular treatments as well (17, 18).

With this limitation of flow diversion recognized, endovascular surgeons began to devise strategies for maximizing occlusion outcomes. These strategies include layering multiple stents and using coils adjunctively (13, 19). In our experience, the latter is more effective, safer, and less costly. In this case, passing the BTO allowed us to feel comfortable that coils could be used, and if the ophthalmic artery were to thrombose, then the patient would be unlikely to experience vision loss. If our case-patient were to have failed the BTO, it would have been taken as evidence that adjunctive coiling imperils vision and that single-modality flow diversion is unlikely to resolve the aneurysm, which would have led to more serious consideration of surgical clipping.

Prior to advancements in endovascular technology, microsurgical clipping has been the treatment modality used to address OphAs (20). Compared to other anterior circulation aneurysms, clipping OphAs is more invasive because of the need for neck dissection to establish proximal control in the event of a rupture and the need to drill the anterior clinoid process to expose the aneurysm neck. In addition to its invasiveness, microsurgical clipping is technically challenging due to the complex surrounding anatomy, including the anterior clinoid, oculomotor nerve (CN3), cavernous sinus, internal carotid artery (ICA) perforating branches, and, most notably, optic nerve (CN1) (21). This technique offers high rates of aneurysm occlusion and low rates of recurrence, though visual morbidity occurs in ~10%–30% of cases (20, 22–26). A series of 345 surgically treated OphAs reiterates this, as microsurgical clipping was found to have higher odds of visual morbidity (OR 8.5, 95% CI 1.1–64.9, p = 0.04) although lower odds of residual and re-treatment (OR 0.06, 95% CI 0.01–0.28, p < 0.001; OR 0.12, 95% CI 0.02–0.58, p = 0.008, respectively) when compared to endovascular therapies (27). Importantly, the definitive nature of surgical clipping may be exaggerated in observational studies, and the non-curative treatment rate, in fact, likely approaches 12% (28).

Several mechanisms of injury to the optic nerve may occur during surgical clipping, for example, thermal injury from high-speed drilling of the anterior clinoid process, ischemic injury from perforator sacrifice, or mechanical trauma from nerve manipulation or clip compression (20). Each of these risks is avoided in an endovascular approach, such as balloon-assisted coiling (i.e., inflating a balloon at the base of the aneurysm during coiling to secure each coil within the aneurysm’s dome), stent-assisted coiling (i.e., deploying a stent at base of the aneurysm to secure the coils within the aneurysm’s dome), flow diversion, or flow diversion with adjunctive coiling. While avoiding the challenges of an open approach is what facilitates lower visual morbidity when treating OphAs endovascularly, the recanalization and recurrence rates are higher (21, 24, 25, 27, 29–31). In a cohort of 138, Lu et al. (21) found a 16.1% vs. 2.4% rate of visual deficits after clipping vs. coiling, respectively, while D’Urso et al. (31) recognized a 17% rate of recurrence and a 9% rate of re-treatment among a series of 74 carotid-ophthalmic aneurysms treated with coiling alone. This is compared to a 97% rate of complete occlusion when treated with clipping (25).

Griessenauer et al. (32) provided a useful classification scheme for OphAs that can be used to decide when BTO and adjunctive coiling may be necessary. The authors describe three classes that are dependent upon the anatomical relationship of the ophthalmic artery’s origin and the aneurysm, as follows: type 1, ophthalmic artery origin entirely separate from the aneurysm; type 2, ophthalmic artery origin is incorporated in the aneurysm’s neck similar to the OphA that we encountered in our case; type 3, ophthalmic artery origin arises from the aneurysm dome. After flow diversion, type 1 OphA demonstrated the highest rate of occlusion (89.5%), whereas type 3 OphAs had the lowest rate of occlusion (50%) and ophthalmic artery patency (85.7%) as well as the highest rate of visual deficits (14.3%) with flow diversion. Though using a different classification scheme, the risk of visual morbidity associated with flow diversion of OphAs, particularly type 3, is also described by Rouchaud et al. (33). These data presented by Rouchaud et al. (33) should be analyzed with caution, as BTO was not utilized to assess risk to vision prior to deployment of the flow diverting stent. Nonetheless, given the morbidity and diminished efficacy of flow diversion for type 3 OphAs, it is critical to assess the morphology of the OphA and distinguish the infundibula from a true aneurysm before proceeding with elective treatment. Type 2 OphAs have higher occlusion rates (70%) than type 3, but also higher rates of visual deficits (8.3%) compared to type 1. For all of these reasons, perhaps it is type 2 ophthalmic aneurysms that best warrant BTO and consideration of flow diversion with adjunctive coiling.

## Conclusion

OphAs remain one of the most complex cerebral aneurysms to manage surgically. Microsurgical clipping offers durability but is limited by visual morbidity, while endovascular approaches may have diminished efficacy with the benefit of improved visual outcomes. Balloon test occlusion of the ophthalmic artery and flow diversion with adjunctive coiling are advanced endovascular techniques that may be employed in specific clinical scenarios to optimize occlusion outcomes while minimizing morbidity.

## Data availability statement

The original contributions presented in the study are included in the article/supplementary material. Further inquiries can be directed to the corresponding author.

## Ethics statement

Written informed consent was obtained from the relevant individual for the publication of any potentially identifiable images or data included in this article.

## Author contributions

TF prepared the manuscript and performed the literature review. TM assisted in the review of the manuscript. ZW assisted in the review of the manuscript and participated in the surgical case. DS assisted in the manuscript review. MB served as the last author, assisted in manuscript preparation, and was the lead surgeon in the case. All authors contributed to the article and approved the submitted version.

## Acknowledgments

The authors would like to acknowledge the support staff in the operating room including the anesthesiologists, nurses, and interventional radiology technicians, whose contributions led to the successful and safe care provided during this case. Additionally, this work was supported by an unrestricted grant to the Ophthalmology Department from Research to Prevent Blindness.

## Conflict of interest

The authors declare that the research was conducted in the absence of any commercial or financial relationships that could be construed as a potential conflict of interest.

## Publisher’s note

All claims expressed in this article are solely those of the authors and do not necessarily represent those of their affiliated organizations, or those of the publisher, the editors and the reviewers. Any product that may be evaluated in this article, or claim that may be made by its manufacturer, is not guaranteed or endorsed by the publisher.
